# Parliament and the Profession

**Published:** 1918-07-20

**Authors:** 


### PARLIAMENT AND THE PROFESSION.

### Medical Treatment of School Children.

The President of the Board of Education, in reply to a question by Sir J. D. Rees, stated that in 1916-17 the total expenditure of local education authorities upon medical inspection and treatment of children in public elementary schools was about £420,000. Mr. Herbert Fisher added that it might be expected that this expenditure will increase after the war in response to the public demand for the improvement and development of the School Medical Service, but any estimate of the ultimate expenditure must be speculative.

### Treatment of Tuberculosis in London.

Mr. Booth asked the President of the Local Government Board if he has received a copy of the resolution passed by the London Insurance Committee in June making certain stipulations as to the practicability of a comprehensive scheme for the treatment of tuberculosis in London, and whether he has summoned a conference of representatives of the committee to meet those of the County Council. Mr. Hayes Fisher's answer was in the affirmative, and he added that the conference was held on July 10.

### Supply of Male Doctors.

Sir A. Geddes informed Mr. Holt that there are at present 5,380 male medical students and 2,250 female medical students at work in the schools and hospitals in the United Kingdom. The male students freshly registered in 1917 numbered 1,378, as against 1,366 in 1914. Administrative arrangements providing for the safeguarding of the supply of male doctors for the next five years are in force, and there does not appear to be any ground for anxiety.

### Invalids' Rations.

Mr. Parker informed Mr. Watt that the new procedure by which Food Control Committees will have power to deal with applications for extra rations in an extended list of diseases, and with applications for white flour in certain cases, would come into force with the introduction of national rationing on July 14. After that date it will only be necessary to refer exceptional cases to the Ministry. Should any cases arise in which life would be endangered by delay, the local committees will have power to deal with them under the special provision made for cases of grave emergency.

### Ministry of Health.

Dr. Addison informed Major Astor that a Bill on the subject of a Ministry of Health is under the consideration of the Government, but he was not able to make any statement as to its introduction.

